# Airborne transmission of respiratory viruses

**DOI:** 10.1126/science.abd9149

**Published:** 2021-08-27

**Authors:** Chia C. Wang, Kimberly A. Prather, Josué Sznitman, Jose L. Jimenez, Seema S. Lakdawala, Zeynep Tufekci, Linsey C. Marr

**Affiliations:** 1 Aerosol Science Research Center, National Sun Yat-sen University, Kaohsiung, Taiwan 804, Republic of China.; 2 Department of Chemistry, National Sun Yat-sen University, Kaohsiung, Taiwan 804, Republic of China.; 3 Scripps Institution of Oceanography, University of California San Diego, La Jolla, CA 92037, USA.; 4 Department of Biomedical Engineering, Israel Institute of Technology, Haifa 32000, Israel.; 5 Department of Chemistry and CIRES, University of Colorado, Boulder, CO 80309, USA.; 6 Department of Microbiology and Molecular Genetics, University of Pittsburgh School of Medicine, Pittsburgh, PA 15219, USA.; 7 School of Information and Department of Sociology, University of North Carolina, Chapel Hill, NC 27599, USA.; 8 Department of Civil and Environmental Engineering, Virginia Tech, Blacksburg, VA 24061, USA.

## Abstract

The COVID-19 pandemic has revealed critical knowledge gaps in our understanding of and a need to update the traditional view of transmission pathways for respiratory viruses. The long-standing definitions of droplet and airborne transmission do not account for the mechanisms by which virus-laden respiratory droplets and aerosols travel through the air and lead to infection. In this Review, we discuss current evidence regarding the transmission of respiratory viruses by aerosols—how they are generated, transported, and deposited, as well as the factors affecting the relative contributions of droplet-spray deposition versus aerosol inhalation as modes of transmission. Improved understanding of aerosol transmission brought about by studies of severe acute respiratory syndrome coronavirus 2 (SARS-CoV-2) infection requires a reevaluation of the major transmission pathways for other respiratory viruses, which will allow better-informed controls to reduce airborne transmission.

## Mechanisms of airborne transmission

The COVID-19 pandemic has highlighted controversies and unknowns about how respiratory pathogens spread between hosts. Traditionally, it was thought that respiratory pathogens spread between people through large droplets produced in coughs and through contact with contaminated surfaces (fomites). However, several respiratory pathogens are known to spread through small respiratory aerosols, which can float and travel in air flows, infecting people who inhale them at short and long distances from the infected person. Wang *et al*. review recent advances in understanding airborne transmission gained from studying the spread of severe acute respiratory syndrome coronavirus 2 (SARS-CoV-2) infections and other respiratory pathogens. The authors suggest that airborne transmission may be the dominant form of transmission for several respiratory pathogens, including SARS-CoV-2, and that further understanding of the mechanisms underlying infection from the airborne route will better inform mitigation measures. —GKA

## Abstract (structured)

**BACKGROUND:**

Exposure to droplets produced in the coughs and sneezes of infected individuals or contact with droplet-contaminated surfaces (fomites) have been widely perceived as the dominant transmission modes for respiratory pathogens. Airborne transmission is traditionally defined as involving the inhalation of infectious aerosols or “droplet nuclei” smaller than 5 μm and mainly at a distance of >1 to 2 m away from the infected individual, and such transmission has been thought to be relevant only for “unusual” diseases. However, there is robust evidence supporting the airborne transmission of many respiratory viruses, including severe acute respiratory syndrome coronavirus (SARS-CoV), Middle East respiratory syndrome (MERS)–CoV, influenza virus, human rhinovirus, and respiratory syncytial virus (RSV). The limitations of traditional views of droplet, fomite, and airborne transmission were illuminated during the COVID-19 pandemic. Droplet and fomite transmission of SARS-CoV-2 alone cannot account for the numerous superspreading events and differences in transmission between indoor and outdoor environments observed during the COVID-19 pandemic. Controversy surrounding how COVID-19 is transmitted and what interventions are needed to control the pandemic has revealed a critical need to better understand the airborne transmission pathway of respiratory viruses, which will allow for better-informed strategies to mitigate the transmission of respiratory infections.

**ADVANCES:**

Respiratory droplets and aerosols can be generated by various expiratory activities. Advances in aerosol measurement techniques, such as aerodynamic and scanning mobility particle sizing, have shown that the majority of exhaled aerosols are smaller than 5 μm, and a large fraction are <1 μm for most respiratory activities, including those produced during breathing, talking, and coughing. Exhaled aerosols occur in multiple size modes that are associated with different generation sites and production mechanisms in the respiratory tract. Although 5 μm has been used historically to distinguish aerosols from droplets, the size distinction between aerosols and droplets should be 100 μm, which represents the largest particle size that can remain suspended in still air for more than 5 s from a height of 1.5 m, typically reach a distance of 1 to 2 m from the emitter (depending on the velocity of airflow carrying the aerosols), and can be inhaled. Aerosols produced by an infected individual may contain infectious viruses, and studies have shown that viruses are enriched in small aerosols (<5 μm). The transport of virus-laden aerosols is affected by the physicochemical properties of aerosols themselves and environmental factors, including temperature, relative humidity, ultraviolet radiation, airflow, and ventilation. Once inhaled, virus-laden aerosols can deposit in different parts of the respiratory tract. Larger aerosols tend to be deposited in the upper airway; however, smaller aerosols, although they can also be deposited there, can penetrate deep into the alveolar region of the lungs. The strong effect of ventilation on transmission, the distinct difference between indoor and outdoor transmission, well-documented long-range transmission, the observed transmission of SARS-CoV-2 despite the use of masks and eye protection, the high frequency of indoor superspreading events of SARS-CoV-2, animal experiments, and airflow simulations provide strong and unequivocal evidence for airborne transmission. Fomite transmission of SARS-CoV-2 has been found to be far less efficient, and droplets are only dominant when individuals are within 0.2 m of each other when talking. Although both aerosols and droplets can be produced by infected individuals during expiratory activities, droplets fall quickly to the ground or surfaces within seconds, leaving an enrichment of aerosols over droplets. The airborne pathway likely contributes to the spread of other respiratory viruses whose transmission was previously characterized as droplet driven. The World Health Organization (WHO) and the US Centers for Disease Control and Prevention (CDC) have officially acknowledged the inhalation of virus-laden aerosols as a main transmission mode in spreading COVID-19 at both short and long ranges in 2021.

**OUTLOOK:**

Airborne transmission of pathogens has been vastly underappreciated, mostly because of an insufficient understanding about the airborne behavior of aerosols and at least partially because of the misattribution of anecdotal observations. Given the lack of evidence for droplet and fomite transmission and the increasingly strong evidence for aerosols in transmitting numerous respiratory viruses, we must acknowledge that airborne transmission is much more prevalent than previously recognized. Given all that we have learned about SARS-CoV-2 infection, the aerosol transmission pathway needs to be reevaluated for all respiratory infectious diseases. Additional precautionary measures must be implemented for mitigating aerosol transmission at both short and long ranges, with particular attention to ventilation, airflows, air filtration, UV disinfection, and mask fit. These interventions are critical tools for ending the current pandemic and preventing future outbreaks.

## Teaser

A Review discusses the scientific basis of and factors controlling airborne transmission of respiratory viruses including coronavirus.

## Graphical abstract

**Figure:**
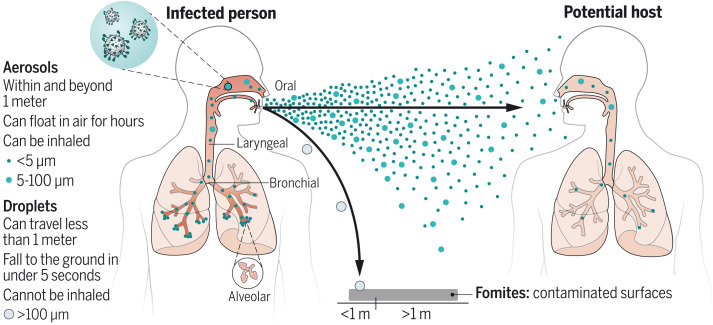
Phases involved in airborne transmission of respiratory viruses. Virus-laden aerosols (<100 I1/4m) are first generated by an infected individual through expiratory activities, through which they are exhaled and transported in the environment. They may be inhaled by a potential host to initiate a new infection, provided that they remain infectious. In contrast to droplets (>100 I1/4m), aerosols can linger in air for hours and travel beyond 1 to 2 m from the infected individual who exhales them, causing new infections at both short and long ranges.

CREDIT: N. CARY/*SCIENCE*

Over the past century, respiratory viruses were thought to be spread mainly through large respiratory droplets, produced in the coughs and sneezes of infected individuals that deposit on the mucous membranes of the eyes, nose, or mouth of potential hosts (droplet transmission) or that deposit on surfaces that are then touched by potential hosts and transferred to mucous membranes (fomite transmission). Such droplets are thought to fall to the ground within 1 to 2 m of the infectious person—a key assumption used by most public health agencies in recommending a safe distance from people infected with respiratory viruses. Thought to be less common, airborne transmission refers to the inhalation of infectious aerosols or “droplet nuclei” (droplets that evaporate in the air), often defined to be smaller than 5 μm and traveling distances of >1 to 2 m away from the infected individual. Aerosols are microscopic liquid, solid, or semisolid particles that are so small that they remain suspended in air. Respiratory aerosols are produced during all expiratory activities, including breathing, talking, singing, shouting, coughing, and sneezing from both healthy individuals and those with respiratory infections (*1*–*4*).

The historical definition of airborne transmission ignores the possibility that aerosols can also be inhaled at close range to an infected person, where exposure is more likely because exhaled aerosols are more concentrated closer to the person emitting them. Moreover, rather than the conventional definition of 5 μm, it has recently been suggested that the size distinction between aerosols and droplets should be updated to 100 μm, as this distinguishes between the two on the basis of their aerodynamic behavior (*5*–*7*). Specifically, 100 μm represents the largest particles that remain suspended in still air for >5 s (from a height of 1.5 m), travel beyond 1 m from the infectious person, and can be inhaled. Although droplets produced by an infectious individual through coughing or sneezing may convey infection at short distances (<0.5 m), the number and viral load of aerosols produced through speaking and other expiratory activities are much higher than those of droplets (*8*–*10*). Aerosols are small enough to linger in air, accumulate in poorly ventilated spaces, and be inhaled at both short and long ranges, calling for an urgent need to include aerosol precautions in current respiratory disease control protocols. During the COVID-19 pandemic, controls have focused mainly on protecting against droplet and fomite transmission, whereas the airborne route has required much more evidence before controls can be added to protect against it.

Debates surrounding the relative importance of different transmission modes in spreading respiratory disease have spanned centuries. Before the 20th century, infectious respiratory diseases were thought to spread by “pestilential particles” released by infected individuals (*11*, *12*). This view of airborne transmission was dismissed in the early 1900s by Charles Chapin, who claimed that contact was the chief route for respiratory disease transmission, with spray-borne (droplet) transmission being an extension of contact transmission (*13*). Chapin was concerned that mentioning transmission by air would scare people into inaction and displace hygiene practices. Chapin erroneously equated infections at close range with droplet transmission—neglecting the fact that aerosol transmission also occurs at short distances. This unsupported assumption became widespread in epidemiological studies (*14*), and mitigation strategies for controlling respiratory virus transmission have since focused on limiting droplet and fomite transmission (*15*). Some of these strategies are also partially effective for limiting aerosol transmission, leading to the erroneous conclusion that their efficacy proved droplet transmission.

Despite the assumed dominance of droplet transmission, there is robust evidence supporting the airborne transmission of many respiratory viruses, including measles virus (*16*–*18*), influenza virus (*19*–*24*), respiratory syncytial virus (RSV) (*25*), human rhinovirus (hRV) (*9*, *26*–*28*), adenovirus, enterovirus (*29*), severe acute respiratory syndrome coronavirus (SARS-CoV) (*30*, *31*), Middle East respiratory syndrome coronavirus (MERS-CoV) (*32*), and SARS-CoV-2 (*33*–*36*) (Table 1). Airborne transmission has been estimated to account for approximately half of the transmission of influenza A virus in one study of a household setting (*20*). A human challenge study on rhinovirus transmission concluded that aerosols were likely the dominant transmission mode (*26*). SARS-CoV-2 infection of hamsters and ferrets has been shown to transmit through air in experimental configurations designed to exclude contributions from direct contact and droplet transmission (*33*, *37*, *38*). Analysis of respiratory emissions during infection with influenza virus, parainfluenza virus, RSV, human metapneumovirus, and hRV has revealed the presence of viral genomes in a variety of aerosol sizes, with the highest amount detected in aerosols <5 μm rather than in larger aerosols (*39*). SARS-CoV-2 RNA has been detected and infectious virus has been recovered in aerosols ranging from 0.25 to >4 μm (*34*, *35*, *40*–*44*). Influenza virus RNA has also been detected in both fine (≤5 μm) and coarse (>5 μm) aerosols exhaled from infected individuals, with more viral RNA contained in the fine aerosol particles (*23*). Laboratory studies have found that aerosolized SARS-CoV-2 has a half-life of ~1 to 3 hours (*45*–*47*). The World Health Organization (WHO) and the US Centers for Disease Control and Prevention (CDC) officially acknowledged inhalation of virus-laden aerosols as a main mode in spreading SARS-CoV-2 at both short and long ranges in April and May of 2021, respectively (*48*, *49*).

**Table 1.** Airborne transmission of respiratory viruses. Representative evidence of airborne transmission for various respiratory viruses and their basic reproduction number. Cells with dashes indicate not applicable.

**Virus name**	**Scope of studies and/or approaches**							**Basic** **reproduction** **number (R_0_)**
	**Air** **sampling** **and PCR**	**Air sampling** **and cell** **culture**	**Animal** **models**	**Laboratory** **or clinical** **studies**	**Epidemiological** **analysis**	**Simulation** **and** **modeling**	**Size-resolved** **information**	
SARS-CoV	(*31*)	(*31*)	–	(*30*)	(*30*)	(*30*)	–	2.0–3.0 (*197*)
MERS-CoV	(*32*)	(*32*, *103*)	(*103*, *198*)	(*32*)	–	–	–	0.50–0.92 (*197*)
SARS-CoV-2	(*41*–*44*)	(*34*, *35*, *40*)	(*33*, *37*, *199*)	(*34*, *45*, *107*)	(*36*, *64*, *71*, *72*, *186*)	(*36*, *50*)	(*34*, *41*, *43*)	1.4–8.9 (*57*, *58*)
Influenza virus	(*22*, *23*, *98*, *102*, *106*)	(*23*, *98*, *101*)	(*24*, *137*, *200*, *201*)	(*24*, *138*, *202*, *203*)	(*20*)	(*20*, *114*, *204*)	(*23*, *105*, *106*)	1.0–21 (*205*)
Rhinovirus	(*9*, *27*)	(*26*, *28*)	–	(*26*–*28*)	–	(*27*)	(*9*)	1.2–2.7 (*205*)
Measles virus	(*16*)	(*16*)	–	–	(*17*)	(*17*)	(*16*)	12–18 (*206*)
Respiratory syncytial virus (RSV)	(*102*)	(*25*)	–	(*25*)	–	–	(*25*)	0.9–21.9 (*205*)

Mathematical modeling of exposure to respiratory pathogens supports that transmission is dominated by short-range aerosol inhalation at most distances within 2 m of the infectious person, and droplets are only dominant when individuals are within 0.2 m when talking or 0.5 m when coughing (*50*). Anecdotal observations of measles virus (*16*–*18*) and *Mycobacterium tuberculosis* (*51*, *52*) infection in close proximity, previously attributed solely to droplets, include transmission by aerosols at short range. Further studies are warranted for respiratory diseases whose transmission has previously been characterized as droplet driven because it is plausible that airborne transmission is important or even dominant for most of them.

Early in the COVID-19 pandemic, it was assumed that droplets and fomites were the main transmission routes on the basis of the relatively low basic reproduction number (R_0_) compared with that of measles (*53*–*55*) (Table 1). R_0_ is the average number of secondary infections caused by a primary infected individual in a homogeneously susceptible population. This argument was built on a long-standing belief that all airborne diseases must be highly contagious. However, there is no scientific basis for such an assumption because airborne diseases exhibit a range of R_0_ values that cannot be well represented by a single average value, which depends on numerous factors. For example, tuberculosis (R_0_, 0.26 to 4.3) is an obligate airborne bacterial infection (*56*), but it is less transmissible than COVID-19 (R_0_, 1.4 to 8.9) (*57*–*59*). The factors affecting airborne transmission include viral load in different-sized respiratory particles, the stability of the virus in aerosols, and the dose-response relationship for each virus (the probability of infection given exposure to a certain number of virions through a particular exposure route). Moreover, R_0_ is an average, and COVID-19 is greatly overdispersed, meaning that, under certain conditions, it can be highly contagious. Epidemiological studies have found that 10 to 20% of infected individuals account for 80 to 90% of subsequent infections for SARS-CoV-2, highlighting the heterogeneity in secondary attack rates (the proportion of exposed individuals who become infected) (*60*–*63*).

A growing body of research on COVID-19 provides abundant evidence for the predominance of airborne transmission of SARS-CoV-2. This route dominates under certain environmental conditions, particularly indoor environments that are poorly ventilated (*6*, *34*, *35*, *41*, *42*, *45*, *50*, *64*–*68*), an observation that implicates solely aerosols because only aerosols—and not large droplets or surfaces—are affected by ventilation. Moreover, the marked difference between rates of indoor and outdoor transmission can only be explained by airborne transmission, because large droplets, whose trajectories are affected by gravitational settling but not ventilation, behave identically in both settings (*69*). Various combinations of epidemiological analyses; airflow model simulations; tracer experiments; and analysis and modeling of superspreading events in restaurants (*36*), in meatpacking plants (*70*), on a cruise ship (*71*), during singing at a choir rehearsal (*64*), and the long-distance transmission at a church (*72*) all implicate aerosols as the most likely mode of transmission over fomites and droplets. It is highly unlikely that most people at any of these events all touch the same contaminated surface or are exposed to droplets produced from the cough or sneeze of an infectious person at close range and encounter sufficient virus load to cause infection. However, the one common factor for all people at these indoor events is the shared air they inhale in the same room. Commonalities among superspreading events include indoor settings, crowds, exposure durations of 1 hour or more, poor ventilation, vocalization, and lack of properly worn masks (*36*). Given that droplet transmission dominates only when individuals are within 0.2 m when talking (*50*) and that transmission of SARS-CoV-2 through contaminated surfaces is less likely (*73*–*75*), superspreading events can only be explained by including aerosols as a mode of transmission.

To establish effective guidance and policies for protecting against airborne transmission of respiratory viruses, it is important to better understand the mechanisms involved. For airborne transmission to occur, aerosols must be generated, transported through air, inhaled by a susceptible host, and deposited in the respiratory tract to initiate infection. The virus must retain its infectivity throughout these processes. In this Review, we discuss the processes involved in the generation, transport, and deposition of virus-laden aerosols, as well as the important parameters that influence these processes, which are critical to informing effective infection control measures (Fig. 1).

**Fig. 1.**
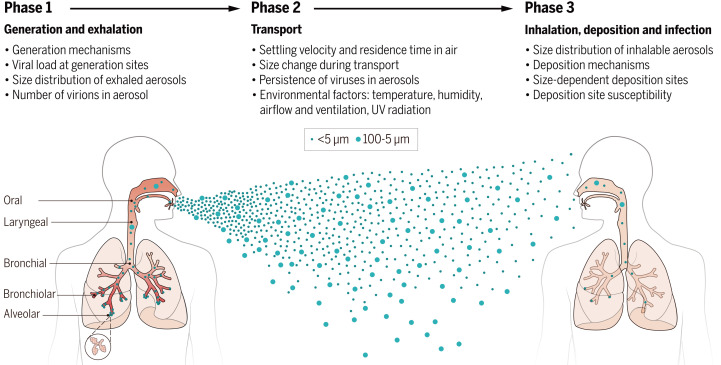
Airborne transmission of respiratory viruses. Phases involved in the airborne transmission of virus-laden aerosols include (i) generation and exhalation; (ii) transport; and (iii) inhalation, deposition, and infection. Each phase is influenced by a combination of aerodynamic, anatomical, and environmental factors. (The sizes of virus-containing aerosols are not to scale.)

## Generation of virus-laden aerosols

**Fig. 2.**
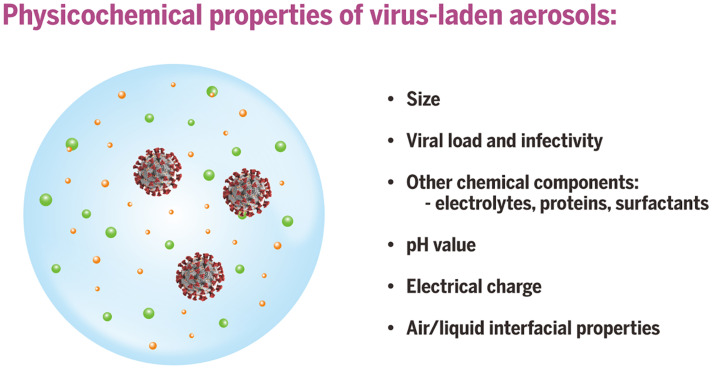
Physicochemical properties of virus-laden aerosols. The behavior and fate of virus-laden aerosols are inherently governed by their characteristic properties, including physical size, viral load, infectivity, other chemical components in the aerosol, electrostatic charge, pH, and the air-liquid interfacial properties.

Expiratory activities produce aerosols from different sites in the respiratory tract through distinct mechanisms. Aerosols produced by activities such as breathing, speaking, and coughing exhibit different aerosol size distributions and airflow velocities (*76*, *77*), which in turn govern the types and loads of viruses that each aerosol particle may carry, the residence time in air, the distance traveled, and ultimately the deposition sites in the respiratory tract of a person who inhales them (*78*). Aerosols released by an infected individual may contain viruses (*39*, *79*–*81*) as well as electrolytes, proteins, surfactants, and other components in the fluid that lines respiratory surfaces (*82*, *83*) (Fig. 2).

### Sites of aerosol formation

Respiratory aerosols can be classified into alveolar, bronchiolar, bronchial, laryngeal, and oral aerosols, according to the sites where they are produced (*3*, *84*, *85*). Bronchiolar aerosols are formed during normal breathing (*3*). During exhalation, the liquid film lining the lumenal surfaces of the bronchioles ruptures to produce small aerosols. Such aerosols are generated by shear forces that destabilize the air-liquid or air-mucous interface. Respiratory airflows are often turbulent under high airflow velocities, particularly in the large lumens of the upper airways, which transition to laminar flow in the bronchi and bronchioles (*76*, *86*–*88*). Laryngeal aerosols are generated through vocal fold vibrations during vocalization (*3*). The apposition of vocal folds forms liquid bridges, which burst into aerosols during exhalation. By contrast, droplets (>100 μm) are primarily produced from saliva in the oral cavity (*3*). Aerosol emission rates increase with airflow velocity and speech volume during activities such as singing and shouting (*9*, *89*, *90*).

### Number and size distributions

The size of exhaled aerosols is one of the most influential properties governing their fate, because size not only determines their aerodynamic characteristics but also their deposition dynamics and the site of infection. Size distributions of respiratory aerosols have been investigated since the 1890s using various approaches, including optical microscopy, high-speed photography, and, more recently, laser-based detection techniques (*1*, *2*, *91*). Early studies used measuring techniques and analytical methods that were unable to detect aerosols <5 μm (*1*, *92*), but current instruments, such as aerodynamic and scanning mobility particle sizing systems, have enabled the detection of smaller aerosols. Respiratory aerosols produce a multimodal size distribution, with peaks around 0.1 μm, 0.2 to 0.8 μm, 1.5 to 1.8 μm, and 3.5 to 5.0 μm, each representing a different generation site, production process, and expiratory activity (*2*, *8*, *9*, *85*, *91*, *93*). The smaller the modal size, the deeper the aerosols originate in the respiratory tract. A larger mode centered at 145 μm for talking and 123 μm for coughing originates mainly from the oral cavity and lips (*3*). In terms of number, the majority of exhaled aerosols are <5 μm, and a large fraction are <1 μm for most respiratory activities, including those produced during breathing, talking, and coughing (*8*, *9*). Overall, speech produces 100 to 1000 times the number of aerosols <100 μm in size for every droplet that is >100 μm (*3*).

Normal breathing has been shown to release up to 7200 aerosol particles per liter of exhaled air (*9*, *93*). The number of virus-laden aerosols expelled by individuals while breathing varies widely between individuals and depends on disease stage, age, body mass index, and preexisting health conditions (*94*, *95*). Children generally produce fewer virus-laden aerosols than adults because their lungs are still developing and have fewer bronchioles and alveoli in which aerosols can form (*96*). The processes involved in aerosol formation, particularly the properties of fluid lining the airways that affect its propensity to break up to form aerosols, plays a crucial role in the number of aerosols exhaled (*94*). One study showed that 1 min of speaking may produce at least 1000 aerosols (*97*). Although coughing can produce more aerosols in a short period of time, it is much more sporadic than continuous breathing and speaking, especially for infected individuals who display no clinical symptoms. Therefore, breathing, speaking, and other continuous vocalization by infected individuals will likely release more total virus-laden aerosols overall than less-frequent coughing.

### Viral content of aerosols

The viral load of aerosols is a key factor in determining the relative contribution of airborne transmission. However, sampling and detecting airborne viruses is challenging because of their low concentrations in air and susceptibility to destruction and inactivation during sampling. Air samples are often analyzed for the presence of viral genomes by quantitative polymerase chain reaction (qPCR) or quantitative reverse transcription PCR (qRT-PCR) methods, which are highly sensitive. Nevertheless, the presence of genetic material alone does not indicate whether the virus is infectious. The viability of viruses depends on the integrity and function of their genomic material, nucleoprotein, capsid, and/or envelope. Although some studies have tried and failed to culture viruses from air, the use of more gentle methods, such as a liquid condensation collection device, has enabled the detection of numerous viable respiratory viruses, including influenza viruses and SARS-CoV-2 in aerosols (*35*, *40*, *98*).

Many viruses have been isolated from breath and indoor air samples, including adenovirus (*29*, *99*), coxsackievirus (*100*), influenza viruses (*22*, *23*, *98*, *101*), rhinovirus (*9*, *26*–*28*), measles virus (*16*, *17*), RSV (*25*, *102*), SARS-CoV (*31*), MERS-CoV (*32*, *103*), and SARS-CoV-2 (*34*, *35*, *40*–*44*) (Table 1). The concentration of SARS-CoV-2 in the air of a hospital room with two COVID-19 patients was between 6 and 74 TCID_50_ per liter (median tissue culture infectious dose per liter) (*35*). The distribution of virions across different sizes of aerosol particles is related to their site of generation, the production mechanism, and the severity of infection at the generation site, which varies among different viruses (*104*). It is commonly assumed that viral concentrations in clinical samples (e.g., sputum or saliva) translate directly to the concentration in droplets and aerosols generated from respiratory fluid—i.e., that viral load scales with the initial volume of droplets and aerosols (*50*, *55*, *71*). However, size-segregated samples of aerosols collected in the exhaled breath of individuals infected with influenza A or B viruses, parainfluenza virus, coronaviruses, hRV, or RSV and air collected in various settings show that viruses are enriched in smaller aerosols (*10*). In samples collected from influenza patients while breathing, talking, and/or coughing, more than half of the viral RNA was found in aerosols <4 to 5 μm (*23*, *104*, *105*). A study of several respiratory viruses found viral RNA more commonly in small (<5 μm) than in large aerosols (*39*). The distribution of influenza virus and RSV in ambient aerosols measured in a medical clinic revealed that 42% of influenza A virus RNA, but only 9% of RSV RNA, was in aerosols ≤4 μm (*102*). In a study that collected aerosols in a health clinic, childcare center, and airplanes, more than half of influenza A virus RNA was found in aerosols <2.5 μm (*106*). A study found that a subset of COVID-19 patients release up to 10^5^ to 10^7^ SARS-CoV-2 genome copies per hour in exhaled breath, whereas others do not exhale detectable virus (*107*). Large interpersonal variability in both the number of aerosols produced and their viral load may contribute to overdispersion in COVID-19 transmission, a crucial component in superspreading events (*108*).

Although infectious viruses are enriched in small aerosols, the dose-response relationship that governs the probability of infection given exposure to a certain number of virions, remains to be determined. In a susceptible host, the minimum infectious dose varies on the basis of virus type and deposition site within the respiratory tract, such that the inhalation of smaller aerosols that deposit deeper in the lungs could require less virus to initiate infection. Studies on influenza virus have shown that the dose required to initiate infection in humans, in terms of plaque-forming units (PFU), is, for the inhalation of aerosols, about a hundredth the size of the dose for intranasal inoculation (*101*). Improved characterization of the viral load and distribution of infectious virions in individual aerosols as a function of particle size, for different people and stages of disease, will greatly contribute to our understanding of airborne transmission of respiratory viruses.

## Virus-laden aerosols in the environment

The physical characteristics of aerosols affect their transport in air. The initial velocity of respiratory aerosols depends on how they are generated within and released from the respiratory tract; for example, coughing produces droplets and aerosols released at higher velocities than speaking (*109*). Aerosol transport is controlled by a combination of airflow and environmental properties and by the physical characteristics of the aerosols themselves. Aerosols may diverge from streamlines as a result of inertia, Brownian motion, and external forces including gravitational, electrophoretic, and thermophoretic forces. Such motions can also lead to removal from air by deposition on surfaces. The lifetime of viruses in air is a function of physical transport and biological inactivation, which are affected by environmental factors, such as temperature, humidity, and ultraviolet (UV) radiation.

The sizes of exhaled aerosols that remain airborne evolve over time as a result of evaporation, coagulation, and/or deposition. Evaporation of water from aqueous aerosols is normally described by the Hertz-Knudsen equation (*110*). However, because respiratory aerosols contain nonvolatile components including proteins, electrolytes, and other biological species, the evaporation rate is slower than that of pure water (*111*). During evaporation, aerosols are subject to changes in phase, morphology, viscosity, and pH, all of which have been studied in simulated but not actual respiratory aerosols (*83*, *112*). Changes in physical characteristics of aerosols will affect the transport and fate of any viruses they contain, and associated changes in chemical characteristics of aerosols can affect virus viability (*113*). The overall size distributions of virus-laden aerosols in air also evolve over time because larger aerosols are preferentially removed by sedimentation to the ground or other surfaces, causing the median of the distribution to shift toward smaller sizes (*114*).

The residence time of virus-laden aerosols in air is crucial in determining their range of spread. In the absence of other forces, the residence time of an aerosol of a specific size is related to its terminal settling velocity, *u*_p_, resulting from a balance between the viscous drag force and the gravitational force, as described by Stokes’ law for small particles subject to laminar flow (*115*, *116*)

$$u_{\text{p}}=\frac{d_{\text{p}}^{2}g\rho_{\text{p}}C_{\text{c}}}{18\eta }$$

where *d*_p_ is the diameter of the aerosol particle, *g* is gravitational acceleration, ρ_p_ is the density of the aerosol particle, *C*_c_ is the Cunningham slip correction factor accounting for the reduced air resistance caused by slippage when the particle size becomes comparable to the mean free path of gas molecules, and η is the dynamic viscosity of air.

**Fig. 3.**
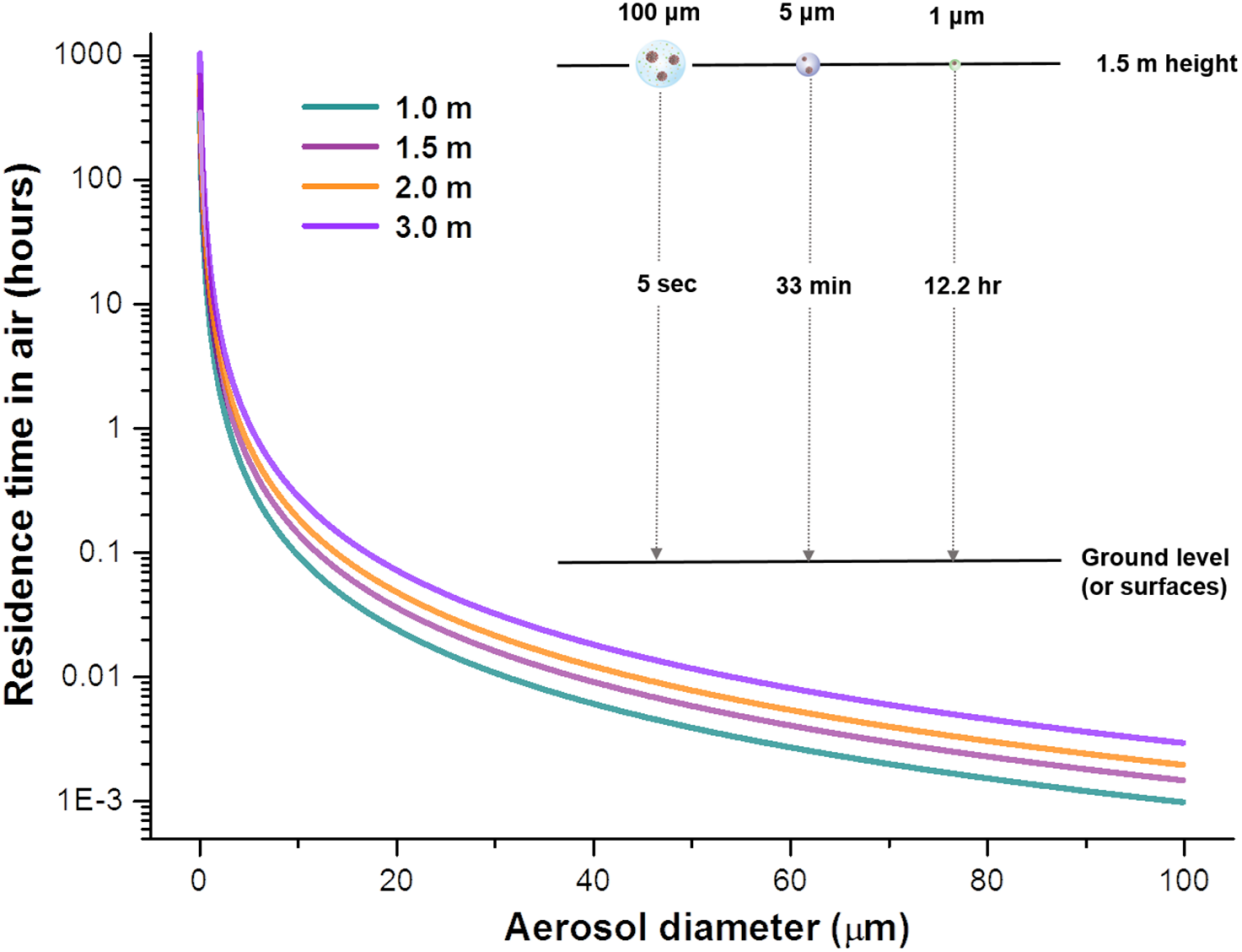
How long can aerosols linger in air? Residence time of aerosols of varying size in still air can be estimated from Stokes’ law for spherical particles (*116*). For example, the time required for an aerosol of 100, 5, or 1 μm to fall to the ground (or surfaces) from a height of 1.5 m is 5 s, 33 min, or 12.2 hours, respectively.

The settling time for aerosols of a specific size to reach the ground can thus be estimated on the basis of an assumption that the surrounding air is at rest (Fig. 3). In still air, a 5-μm aerosol takes 33 min to settle to the ground from a height of 1.5 m, whereas a 1-μm aerosol can remain suspended in air for >12 hours (*116*). However, in most realistic environments, the velocity of the surrounding airflow should be taken into consideration. Additionally, when respiratory aerosols are exhaled, these particles are contained in an exhaled humid plume with its own speed and trajectory, which also play a role in determining the final reachable distance and direction (*86*). The distance that virus-laden aerosols travel depends on aerosol size, initial velocity of the flow carrying them, and other environmental conditions, such as outdoor wind speed or indoor air currents induced by natural ventilation or heating, ventilation, and air conditioning (HVAC) systems (*117*, *118*). The concentration of exhaled aerosols is highest close to the source (i.e., the infectious individual) and decreases with distance as the respiratory plume mixes with ambient air (*50*, *119*).

The trajectory and evaporation of exhaled aerosols generated during coughing and speaking have been studied with computational modeling (*117*, *120*). Large droplets tend to reach their maximum horizontal distances quickly and fall to the ground or surfaces within a few meters, whereas aerosols can remain suspended for many seconds to hours, travel long distances, and accumulate in air in poorly ventilated spaces (*117*). The multiphase nature of virus-laden aerosol flows greatly affects flow dynamics and how far aerosols travel, especially for exhalations with higher airflow velocities, such as in a cough (*121*).

## Environmental factors that affect aerosol transmission

Survival of viruses in aerosols, also known as persistence, stability, or retention of infectivity, is commonly determined experimentally using a rotating drum, which allows the aerosols to remain suspended longer than in a stationary chamber. The decay of the virus can be described by first-order kinetics

*C* = *C*_o_ × *e*^−^*^kt^*

where *C* is the concentration of infectious viruses at time *t*, *C*_o_ is the initial concentration of infectious viruses, and *k* is the inactivation rate constant (*122*). The inactivation rate constant differs by virus and depends on a number of factors, including temperature, humidity, UV radiation, and chemical composition of the fluid from which the virus was aerosolized (*45*, *46*, *123*). This dependence, especially on respiratory fluid composition, makes it challenging to compare results across different studies. The time needed to reach 99.99% inactivation varies from hours to months (*124*). The decay rate can be quantified in terms of the half-life, which is ~1 to 3 hours for SARS-CoV and SARS-CoV-2 in laboratory-generated aerosols (*125*–*127*).

### Temperature

Temperature is critical in mediating the survival and transmission of viruses in aerosols (*125*, *128*, *129*), likely by affecting the stability of the proteins, lipids, and genetic material that make up the virus. The upper respiratory tract is maintained at a few degrees cooler than the lungs (*130*), suggesting an enhanced replication capacity in the upper respiratory tract (*131*). SARS-CoV (*132*), SARS-CoV-2 (*133*), and influenza virus (*134*) are more stable at lower temperatures, possibly because of slower decay rates (as governed by the Arrhenius equation) and stronger ordering of phospholipids for enveloped viruses. Epidemiological evidence and animal studies suggest that the transmission of respiratory viruses known to infect the upper airways is favored at lower temperatures (*128*, *135*).

### Relative humidity

By modulating the evaporation rate and equilibrium size of aerosols, relative humidity (RH) affects their transport and the viability of viruses they contain (*113*, *114*, *129*). Respiratory aerosols undergo evaporation upon release from the respiratory tract into ambient air as they transition from a saturated environment to lower RH. The evaporation process is expected to take seconds (*114*, *136*). At lower ambient RH, evaporation occurs more quickly and equilibrates at a smaller equilibrium size (*136*). At RH below ~80%, respiratory aerosols reach a final diameter that is 20 to 40% of the original size (*129*).

The seasonality of cases of influenza virus, human coronaviruses that cause common colds, RSV, and others has been at least partially attributed to RH (*134*). The sensitivity of a virus to RH may be influenced by RH-related effects on virus persistence in the environment and/or immune defenses. Mucociliary clearance is not as efficient at low RH (*134*). Animal studies have shown that influenza virus transmission is favored at low RH (*135*, *137*); however, a study of the 2009 pandemic influenza A virus (H1N1) in more physiologically realistic medium reported that the virus remained highly stable and infectious over a broad RH range between 20 and 100% (*138*). A study investigated the sensitivity of 11 airborne viruses to RH and found that although some RNA viruses survived best at low RH, other viruses survived better at high RH (*139*). The relationship between RH and virus viability in droplets and aerosols is characteristic to the virus, modulated by both the intrinsic physicochemical properties of the virus and its surrounding environment (*113*, *129*, *139*) (Fig. 2).

### UV radiation

Irradiation with UV light has long been established as an effective approach to inactivate airborne viruses, including influenza virus (*127*, *140*), SARS-CoV, and other human coronaviruses (*141*). UV radiation rapidly inactivates SARS-CoV-2 in bulk culture medium (*142*) and in aerosols (*47*) at wavelengths found in ground-level sunlight. UV radiation damages genetic material, leading to inactivation of the virus (*143*). Nevertheless, caution must be taken during operation of UV disinfection lamps to avoid direct eye and skin contact.

### Airflow, ventilation, and filtration

Airflow strongly influences the transport of virus-laden aerosols (*81*) in contrast to droplets, which are rapidly deposited because of gravity. Aerosols in exhaled air tend to rise because the exhaled air is warmer than the environment (*50*), and their trajectories can also be influenced by the body’s thermal plume (*81*). Greater airflow outdoors contributes to greater dispersion, whereas indoors the airflow is restricted by the surrounding walls and ceiling. Ventilation rate and airflow patterns play an important role in airborne transmission of viruses in indoor environments (*144*–*146*). A study of rhinovirus transmission showed that a low ventilation rate increases the risk of exposure to virus-laden aerosols indoors (*27*, *28*). An outbreak of COVID-19 in a high-rise apartment building occurred along vertically aligned units that were connected by a single air duct, demonstrating the risk of airborne transmission associated with shared air (*147*). Improving ventilation rates to reduce the carbon dioxide levels in under-ventilated buildings from 3200 parts per million (ppm) to 600 ppm (corresponding to an estimated increase of ventilation rate from 1.7 liters per second per person to 24 liters per second per person) has been shown to reduce the secondary attack rate of tuberculosis to zero (*146*).

**Fig. 4.**
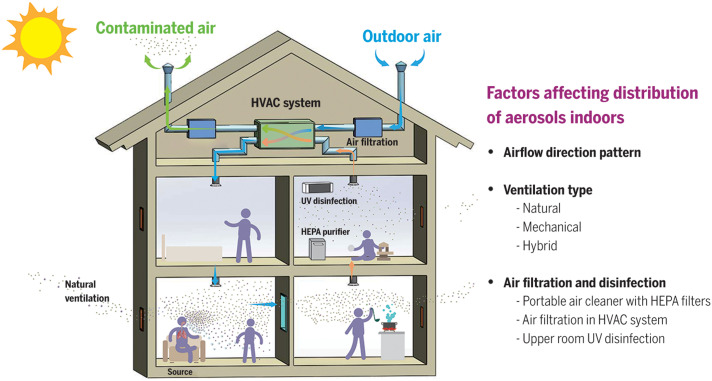
Factors affecting indoor airborne transmission. Whereas the motion of large droplets is predominantly governed by gravity, the movement of aerosols is more strongly influenced by airflow direction and pattern, type of ventilation, and air filtration and disinfection.

The airflow in indoor environments is mediated by the design and operational status of ventilation systems, including the type of ventilation system (whether natural with open windows and doors, mechanical with blowers, or a hybrid of these), airflow patterns, air change rate, and supplementary systems such as air filtration (*145*, *148*) (Fig. 4). The WHO has recently recommended a ventilation rate of 10 liters per second per person (*149*). Proper placement of portable high-efficiency particulate air (HEPA) purifiers, which are capable of removing ≥99.97% of aerosol particles ≥0.3 μm, is also effective in reducing exposure of infectious aerosols, especially when combined with ventilation and universal masking (*150*–*152*). Although ventilation and filtration help to remove virus-laden aerosols, they must be implemented correctly to reduce the spread and risk of aerosol inhalation (*93*, *151*). A study quantitatively assessed the risk of airborne transmission of COVID-19 by asymptomatic individuals in elevator, classroom, and supermarket settings by combining in situ measurements and computational fluid dynamics (CFD) simulations, showing that inappropriate ventilation may create hotspots with risks much higher than in other room locations (*93*). Additionally, the physical plexiglass barriers designed to block droplet spray from coughs and sneezes in indoor spaces can impede the airflow and even trap higher concentrations of aerosols in the breathing zone and has been shown to increase transmission of SARS-CoV-2 (*153*).

The risk of airborne infection and correlation with ventilation rate can be assessed by a box model of virus transport and the Wells-Riley infection model (*17*, *64*)

$$P=\frac{N}{S}=1-e^{-Iqpt/Q}$$

where *P* is the probability of infection, *N* is the number of confirmed infection cases, *S* is the number of susceptible cases, *I* is number of infectors, *q* is the quanta (infectious dose) generation rate (quanta per hour), *p* is the pulmonary ventilation rate of susceptible individual (cubic meters per second), *t* is the exposure time (hours), and *Q* is the room ventilation rate (cubic meters per second). A model using the Wells-Riley method was applied to a large community outbreak of COVID-19 in a choir practice with one index case known to be symptomatic that led to 53 cases among 61 members in attendance (87% secondary attack rate), which concluded that poor ventilation along with a crowded venue, loud vocalization, and long duration all contributed to the high secondary attack rate (*64*). The choir practice had limited face-to-face interaction and strong attention on hand disinfection, which allowed major contributions from fomite or droplet transmission to be ruled out (*64*). Research is needed to establish minimum acceptable ventilation rates under different conditions and the effect of ventilation type on the risk of transmission.

## Deposition of virus-laden aerosols

Once inhaled, virus-laden aerosols may deposit in the respiratory tract of a potential host. The size of aerosols is again central to determining the deposition site, although numerous anatomical, physiological, and aerodynamic factors (including the airway anatomical structure, breathing patterns, aerosol transport aerodynamics in the respiratory tract, and the physicochemical properties of inhaled aerosols) also affect the deposition pattern. Infection may be initiated at the deposition site if the virus remains infectious and appropriate receptors are present.

**Fig. 5.**
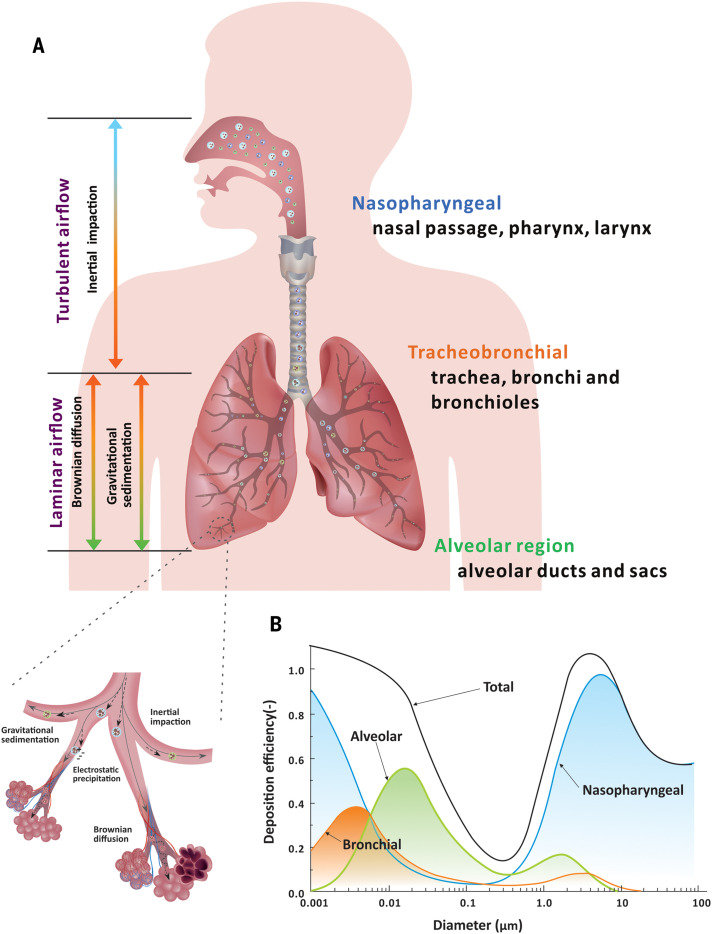
Size-dependent aerosol deposition mechanisms to sites in the respiratory tract. (**A**) Main deposition mechanisms and corresponding airflow regimes in different regions of the human respiratory tract. Large aerosols tend to deposit in the nasopharyngeal region as a result of inertial impaction, whereas small aerosols tend to deposit in the tracheobronchial and alveolar regions on the basis of gravitational sedimentation and Brownian diffusion. An enlarged view of tracheobronchial and alveolar regions illustrates the deposition mechanism. (**B**) The deposition efficiency of aerosols at different regions of the respiratory tract as a function of aerosol diameter based on the ICRP lung deposition model is shown (*116*). The majority of large aerosols deposit in the nasopharyngeal region; only aerosols that are sufficiently small can reach and deposit in the alveolar region.

Aerosols up to 100 μm can be inhaled. Depending on their size, they deposit in different regions of the respiratory tract, based on one of several key mechanisms, including inertial impaction, gravitational sedimentation, Brownian diffusion, electrostatic precipitation, and interception (*154*, *155*) (Fig. 5A). Upon inhalation, the size of inhaled aerosols may increase as a result of hygroscopic growth in the nearly saturated respiratory tract (*156*). The International Committee for Radiological Protection (ICRP) has developed a model, based on human lung architecture, that quantifies deposition efficiency as a function of aerosol size (*157*) (Fig. 5B). Aerosols >5 μm deposit primarily in the nasopharyngeal region (87 to 95%), mainly through inertial impaction and gravitational sedimentation (*115*); although aerosols <5 μm also deposit there, they also may penetrate more deeply into the lungs and deposit in the alveolar lumen (*115*, *157*, *158*). Brownian diffusion is the dominant deposition mechanism of inhaled particles <0.1 μm in the bronchiolar and alveolar regions (*78*, *116*, *159*). Aerosols that carry natural electrostatic charge may be attracted to the airway walls (*160*). Provided a cellular receptor is present at the deposition site, infection may be initiated. The infection efficiency is further governed by the distribution of cellular receptors along the respiratory tract and the virus-host interaction.

Deposition of aerosols in diseased lungs may differ from that in normal lungs because of airway surface structure changes and obstruction by mucous (*161*). Changes in the surface properties of the respiratory epithelium in asthmatic airways and airway narrowing as a result of chronic obstructive pulmonary disease (COPD) alter the airflow and aerodynamic behaviors of inhaled aerosols, thus modifying their deposition dynamics and sites (*162*, *163*). Deposition is generally higher in patients with COPD than in healthy individuals; bronchial deposition is higher in patients with asthma and chronic bronchitis (*154*).

Because viruses are enriched in small aerosols (<5 μm), they can travel deeper into and be deposited in the lower respiratory tract. The viral load of SARS-CoV-2 has been reported to be higher and the virus persists longer in the lower respiratory tract compared with the upper respiratory tract (*164*, *165*). Initiation of an infection in the lower respiratory tract adds technical challenges in diagnosing patients because current screening commonly collects samples from the nasopharyngeal or oral cavity using swabs.

## Discussion

Airborne transmission has long been an under-appreciated route for contributing to the transmission of respiratory viral diseases, largely because of an insufficient understanding of the generation and transport processes of virus-laden aerosols as well as misattribution of anecdotal observations. The epidemiological evidence for the dominance of airborne spread of SARS-CoV-2 has increased over time and has become especially strong. First, the distinct difference between indoor and outdoor transmission cannot be explained by droplet transmission because gravity-driven droplets behave identically indoors and outdoors. The high frequency of indoor superspreading events relative to those outdoors points to the importance of airborne transmission (*63*). The demonstrated role of poor ventilation in transmission and superspreading clusters indoors is also only compatible with aerosols, because droplets and fomite transmission are not affected by ventilation. Long-range airborne transmission of SARS-CoV-2 has been observed in hotel quarantines in countries with very low transmission (*166*) and in a large church (*72*).

During the emergence of novel respiratory viruses, a more holistic approach that acknowledges all modes of transmission (airborne, droplet, and fomite) is needed to successfully mitigate risk and prevent spread. The requirement for direct evidence of infectiousness of sampled aerosols before acknowledging and adding controls to address airborne transmission leaves people at potential risk (*69*). When unburdened by conventional definitions of transmission routes, the available evidence for SARS-CoV-2, influenza virus, and other respiratory viruses is much more consistent with transmission by aerosols <100 μm rather than by rare, large droplets sprayed onto mucous membranes of people in very close proximity. Recent acknowledgement of airborne transmission of SARS-CoV-2 by the WHO (*48*) and US CDC (*49*) reinforces the necessity to implement protection against this transmission route at both short and long ranges.

Once the mechanisms leading to airborne transmission are fully understood—acknowledging that transmission by aerosols is largest at close range—it becomes clear there is an overlap in precautions and mitigation measures for both droplets and aerosols (such as distancing and masks), but extra considerations must be taken into account for mitigating aerosol transmission at both short and long ranges. These include attention to ventilation, airflows, mask fit and type, air filtration, and UV disinfection, as well as distinguishing measures between indoor and outdoor environments. Although our knowledge is still increasing, enough is already known to add protective measures to better protect against airborne transmission of respiratory viruses, noting that “droplet precautions” are not replaced but instead expanded.

A high proportion of individuals infected with SARS-CoV-2 have no symptoms at the time of testing (*167*, *168*). About 20 to 45% of individuals infected with SARS-CoV-2 remained asymptomatic throughout the course of infection, whereas some infected individuals experienced a presymptomatic phase and began to develop symptoms several days after infection (*168*, *169*). The infectiousness of SARS-CoV-2 peaks two days before and extends to one day after symptom onset (*170*). High asymptomatic infection rates have also been reported for influenza virus and other respiratory virus infections (*171*–*173*). Although some studies suggest that airborne transmission is not an efficient route, particularly for asymptomatic and mildly symptomatic individuals who likely have low viral loads in their saliva (*55*), the viral load in presymptomatic individuals is comparable to that of symptomatic patients (*174*, *175*). It is important to implement controls that protect against exposure of infectious virus-laden aerosols produced when infected individuals without any symptoms speak, sing, or simply breathe. Because these individuals do not know they are infected, they generally continue to be involved in social activities, leading to airborne transmission.

Universal masking is an effective and economical way to block virus-laden aerosols (*67*). Model simulations show that masks effectively prevent asymptomatic transmission and reduce the total number of infected individuals as well as mortalities as a result of COVID-19 (*176*). It is crucial to optimize the allocation of masks (*177*). Surgical masks have been shown to reduce the release of influenza virus, seasonal human coronaviruses, and rhinovirus in aerosols <5 μm into the air by infected individuals by up to 100% (*104*, *178*), although for some individuals there was no reduction; and masks are more effective for limiting droplets (*179*). Masks made of combinations of different fabrics and/or multiple layers, when worn properly with no leaks, can block up to 90% of particles between 0.5 and 10 μm (*179*). Small gaps between the mask material and skin can lead to substantial decreases in the overall filtration efficiency. For aerosols <2.5 μm, filtration efficiency decreases by 50% for a relative leak area of 1% (*180*). A study compared the viral filtration efficiency of N95, surgical, and fabric masks using a model virus and found that the efficiency of N95 and some surgical masks exceeded 99%; all fabric masks tested were at least 50% efficient (*181*). The effectiveness of N95, surgical, and cotton masks in blocking SARS-CoV-2–containing aerosols has been investigated using manikins placed face-to-face. N95 respirators demonstrated the highest efficiency in blocking infectious SARS-CoV-2 (*182*). Almost all masks offer at least some protection, but they are not 100% effective. Transmission of SARS-CoV-2 has occurred in health care settings despite medical masks (designed for droplets not aerosols) and eye protection (*183*–*185*), which illustrates the need for proper personal protective equipment (PPE) and layering multiple interventions against airborne transmission, especially in high-risk indoor settings.

Health care facilities are more likely to accommodate patients infected with respiratory viruses. Thus, health care personnel should be provided with proper PPE to reduce airborne exposure. People occupying indoor spaces have increased potential to be exposed to high concentrations of virus-laden aerosols, especially in poorly ventilated and/or crowded indoor settings where virus-laden aerosols can readily accumulate (*93*). Preventive measures should be implemented at all times when traveling in airplanes, trains, buses, ships, and cruise ships, which have relatively small and enclosed air spaces where the ventilation may not always be optimal. Many studies indicate that the risk of airborne transmission in outdoor environments is substantially lower than indoor environments (*186*); however, the risk of transmission outdoors exists in close proximity situations, especially if talking, singing, or shouting over time. The risk of outdoor transmission may rise with increased lifetime and transmissibility of viruses, such as certain variants of SARS-CoV-2 (*187*, *188*). Aerosolization of virus-containing wastewater and hospital fecal discharges also poses potential outdoor exposure risks, which should not be underestimated (*189*).

Implementing effective ventilation systems reduces airborne transmission of infectious virus-laden aerosols. Strategies such as ensuring sufficient ventilation rates and avoiding recirculation are advised (*190*, *191*). Carbon dioxide sensors can be used as indicators of the build-up of exhaled air and serve as a simple way to monitor and optimize ventilation (*192*, *193*). Aerosol sensors can also be used to assess HEPA and HVAC aerosol filtration efficiencies, which are key to lowering infections caused by virus-laden aerosols. Assuring a minimum ventilation rate of 4 to 6 air changes per hour (ACH) and maintaining carbon dioxide levels below 700 to 800 ppm have been advised, although the ventilation type and airflow direction and pattern should also be taken into account (*148*, *194*). Increasing the efficiency of air filtration in HVAC systems, stand-alone HEPA purifiers, or implementing upper room UV disinfection systems can further reduce the concentrations of virus-laden aerosols (*47*, *127*, *140*, *141*, *195*).

Physical distancing, a mitigation put in place to address droplet transmission, is also effective in reducing the chances of aerosol inhalation because aerosol concentrations are much higher in close proximity to an infected individual (*50*). The WHO and many national public health agencies recommend maintaining physical distances of either 1 or 2 m. However, this distance is not sufficient to protect against aerosols that travel beyond this range. If large droplets dominated transmission, distancing alone would have effectively suppressed the transmission of SARS-CoV-2. As has been repeatedly shown in superspreading events, airborne transmission occurs in poorly ventilated rooms when occupants inhale infectious room air (*18*, *36*, *62*, *64*, *71*). Additionally, although distancing helps by moving people away from the most concentrated parts of respiratory plumes, distancing alone does not stop transmission and is not sufficient without accounting for other measures, such as ventilation and filtration, the number of people emitting infectious aerosols, and the amount of time spent in enclosed spaces (*196*). The unknown number of asymptomatic (including presymptomatic) infected individuals present in specific environmental settings is an additional challenge in respiratory disease control. Engineering measures to reduce aerosol concentrations through ventilation, filtration, and upper room UV disinfection remain critical strategies for reducing airborne transmission risks.

Despite the emerging recognition of airborne transmission of respiratory viruses, numerous issues require further exploration. For example, direct measurements are needed of the concentration of virus in aerosols and droplets as a function of size and their potential to initiate a new infection. The lifetime of viruses in aerosols of varying size requires systematic investigation. More studies are needed to quantify the relationship between viral dose delivered by aerosols and droplets and severity of infection; this relationship likely varies considerably for different viruses. It is also important to investigate whether the severity of disease correlates with the size and number of aerosols and the location in which they are deposited in the respiratory tract. Although more studies are needed, unequivocal evidence indicates that airborne transmission is a major pathway for the spread of SARS-CoV-2 and many other respiratory viruses. Additional precautionary measures must be implemented for mitigating aerosol transmission at both short and long ranges, with a major focus on ventilation, airflows, air filtration, UV disinfection, and mask fit. These interventions are critical strategies for helping end the current pandemic and preventing future outbreaks. It is important to note that these proposed measures to improve indoor air quality will lead to long overdue improvements that have health benefits extending well beyond the COVID-19 pandemic.
